# Memory function in opioid-dependent patients treated with methadone or buprenorphine along with benzodiazepine: longitudinal change in comparison to healthy individuals

**DOI:** 10.1186/1747-597X-4-6

**Published:** 2009-04-17

**Authors:** Pekka Rapeli, Carola Fabritius, Hely Kalska, Hannu Alho

**Affiliations:** 1 Unit for Drug Dependence, Department of Psychiatry, Helsinki University Central Hospital, Helsinki, Finland; 2 National Institute for Health and Welfare, Helsinki, Finland; 3 Department of Psychology, Faculty of Behavioural Sciences, Helsinki, Finland; 4 Research Unit of Substance Abuse Medicine, University of Helsinki, Helsinki, Finland

## Abstract

**Background:**

Opioid-substitution treatment (OST) for opioid dependence (OD) has proven effective in retaining patients in treatment and reducing illegal opiate abuse and crime. Consequently, the World Health Organization (WHO) has listed the opioid agonists methadone and buprenorphine as essential drugs for OD that should be available worldwide. In many areas of the world, OD is often associated with concomitant benzodiazepine (BZD) dependence and abuse, which complicates treatment. However, possible changes in the cognitive functioning of these patients are not well-known. The present study is the first to examine longitudinal stability of memory function in OST patients with BZD use, thus providing a new tool for health policy authorities in evaluating the usefulness of OST.

**Methods:**

Within the first two months (T1) and between 6–9 months (T2) after OST admission, we followed the working memory, immediate verbal memory, and memory consolidation of 13 methadone- and 15 buprenorphine- or buprenorphine/naloxone-treated patients with BZD dependence or abuse disorder. The results were compared to those of fifteen normal comparison participants. All participants also completed a self-reported memory complaint questionnaire on both occasions.

**Results:**

Both patient groups performed statistically significantly worse than normal comparison participants in working memory at time points T1 and T2. In immediate verbal memory, as measured by list learning at T1, patients scored lower than normal comparison participants. Both patient groups reported significantly more subjective memory problems than normal comparison participants. Patients with more memory complaints recalled fewer items at T2 from the verbal list they had learned at T1 than those patients with fewer memory complaints. The significance of the main analyses remained nearly the same when the statistical tests were performed without buprenorphine-only patients leaving 12 patients to buprenorphine/naloxone group.

**Conclusion:**

Working memory may be persistently affected in OST patients with BZD use. A high number of memory complaints among OST patients with BZD use may indicate memory consolidation impairment. These findings show that recovery of memory function in OD patients treated along with BZDs takes time, and their memory complaints may have practical relevance.

## Introduction

Opioid-substitution treatment with the full mu opioid receptor agonist methadone or the partial agonist buprenorphine is the most effective treatment for OD [1,2]. Follow-up studies of OST patients have shown consistently high retention in OST, fewer crimes, reduction in substance abuse, and improved health [3,4]. However, the psychosocial recovery of OD patients during treatment is still controversial. It has been stated that while opioid abuse and other problem behavior reduces during the OST, there is little research-based evidence for improvement if patient-centered indicators of quality of life are used [5]. While this critique underestimates the importance of reduction of the health hazards of OD, it also shows the shortage studies using objective measures of psychological functions. In order to meet this challenge, studying memory function of OD patients is an important element, because the patients often complain poor memory [6,7]. Therefore, in this longitudinal study memory function of OST patients was evaluated by tests and subjective memory questionnaire. Because in Finland most OD patients are prescribed benzodiazepines or abuse them from illegal sources [7,8], we examined memory function of this clinically relevant majority.

Some studies have shown substantial memory deficits among OD patients in methadone treatment even after years of treatment [9,10]. Also, buprenorphine-treated patients may show poor memory function [11,12]. However, only two studies have examined the longitudinal stability of memory function during OST. In the seminal longitudinal study by Grevert et al., the memory performance of OST patients, of whom about one third tested positive for other drugs of abuse during the tests, was assessed three times within the first three months of treatment [13]. No baseline or subsequent differences between the methadone patients and a comparison group were seen in objective or subjective memory function. No significant correlations were seen between drug screen status and memory test results. However, as the patients performed the tests immediately before or after the methadone dose, that is, when their plasma concentration is known to be at the lowest level, short-term negative effects of high methadone concentrations may have been missed. In a more recent study by Gruber et al., the tests were done a few hours after the methadone dose [14]. The patients' memory performance was tested first within the first few weeks of OST and again after two months of treatment. Although 65% of the patients tested positive for any illicit use at the first test and 76% at the second test, the results showed a statistically significant improvement in verbal list learning among patients.

In our previous study, we found that both methadone- and buprenorphine/naloxone-treated patients in early OST performed worse than normal comparison participants on a working memory task [15]. The verbal memory deficit was more pronounced in methadone-treated patients than in buprenorphine/naloxone-treated patients. Although the results partially favored buprenorphine/naloxone-treated patients, BZD co-medication that was common in both patient groups, may have affected the results. There are no longitudinal studies comparing the effects of OST drugs while patients use BZDs. However, there is some evidence for acute negative effects of opioid agonists on working memory in drug-naïve healthy volunteers and for chronic negative effects in pain patients [16,17]. The negative effects of BZDs on working memory and long-term memory are better – known, vary from small to moderate, and may last several months after cessation of use [18]. Of special interest is the study of Lintzeris et al., which found that in comparison to a placebo condition, methadone dose alone, or buprenorphine dose in combination with BZD diazepam impairs verbal recall in OST patients [19]. Given these findings suggesting memory deficits in OST patients using BZDs, we did a follow-up study of memory function in OD patients treated with methadone or buprenorphine (including buprenorphine/naloxone) along with BZDs. In order to control for the effects of repeated testing, a comparison group performed similar tests. Working memory, immediate verbal memory, and memory consolidation were examined. The participants also completed the Memory Complaint Questionnaire, which assesses subjective memory function [20].

We hypothesized that working memory function in both OST patient groups treated along with BZDs would be impaired relative to normal comparison participants in the first testing (T1) and would not show improvement. Second, we hypothesized that immediate verbal memory would be impaired relative to normal comparison participants at T1 and would not show improvement in OST patients also using BZDs. Third, we hypothesized that memory consolidation would be impaired in OST patients. Finally, we hypothesized that among OST patients subjective and objective memory function would correlate negatively.

## Methods

The study participants with OD were volunteers admitted for standard OST in the addiction clinics of the Helsinki area. Normal comparison participants were recruited from adult education centers and by word of mouth. All participants included in the study were between 18 – 50 years of age and native Finnish speakers. For OST patients, additional inclusion criteria were OD diagnosis, BZD dependence or abuse diagnosis, start of OST during the last two months, and treatment of OD with methadone, buprenorphine, or buprenorphine/naloxone. We excluded participants with uncontrolled polysubstance abuse, acute alcohol abuse, or acute axis I psychiatric morbidity other than substance abuse related. We also excluded participants with severe brain injury, chronic neurological disease, history of other than substance-induced psychoses, epileptic seizures, human immunodeficiency virus (HIV) infection, pregnancy, or primary cognitive deficit. For these purposes, psychiatric interviews by clinical psychiatrist were conducted for all participants, and diagnostic criteria from the Diagnostic and Statistical Manual of Mental Disorders (DSM-IV) were applied [21,22].

Each OST participant eligible for our study was screened by urine sample for substance abuse on the day of testing and at least once in the preceding month. One third of normal comparison participants were chosen at random for screening for drug of abuse. Participants showing signs of current intoxication, ongoing binge on any substance of abuse, and those with any extra psychoactive drug dose within 24 h were all excluded. According to these criteria 13 methadone- and 15 buprenorphine/naloxone- or buprenorphine-treated patients and 15 normal comparison participants could be studied twice. This represents 59% of volunteer methadone patients at T1, 52% of buprenorphine patients (including both products), and 79% of normal comparison participants. Eight volunteer patients were excluded from the study based on their substance abuse before the test. Fourteen eligible patients and four normal comparison participants dropped out of the study between T1 and T2. At T1, 23% of the methadone patients and 40% of the buprenorphine patients were tested in inpatient settings. At T2, none of the methadone patients and 13% of the buprenorphine patients were tested in inpatient settings.

### Ethics

The study was approved by the independent Hospital District of Helsinki and Uusimaa Ethical Committee (permission 90/2001). The study was conducted in accordance with the 1964 Declaration of Helsinki. All patients were required to be able to read and understand the patient information sheet and sign the informed consent form. All participants were free to discontinue participation in the study whenever they wanted. The participants were reimbursed with € 40 if they attended all study visits.

### Procedure

Cognitive testing was done three to six hours after administration of the opioid-substitution drug. During this time the drug plasma concentration is at its peak [23]. At T1, the methadone patients were given, under supervision on the morning of the test day, a mean dose of 72.9 mg (*SD *= 35.2) of liquid methadone, range 35 – 150 mg. At T2, the respective values for methadone were 125.7 mg (*SD *= 35.8), range 70 – 180 mg. At T1, the buprenorphine patients were given, under supervision on the morning of the test day, a mean dose of 17.3 mg (*SD *= 3.6) of sublingual buprenorphine, range 12 – 24 mg. At T2, the respective values were 22.7 mg (*SD *= 2.9), range 16 – 28 mg. Rise of the dose was statistically significant in both groups (Wilcoxon's Signed Ranks test, exact (2-sided) *p *= 0.001 in both groups). In the buprenorphine group, 80% of the patients were given buprenorphine/naloxone; thus, they were also given sublingual naloxone in a ratio of 1:4 together with their buprenorphine dose. Several studies have shown that among OD individuals sublingual naloxone has minimal if any interference with the opioid agonist effects of the buprenorphine [24-26]. Other prescribed psychoactive medications were given to the patients according to their clinical dose regimen. Table 1 describes the BZDs and their doses used within the 24-hour period before the tests. In order to compare the BZD doses of the groups, all BZDs were converted to diazepam equivalent doses according to their known clinical potency [27]. Temazepam and midazolam doses were halved in order to account for their use as hypnotics on the night before testing. After this conversion, no statistically significant difference existed between the patient groups in their mean estimated diazepam equivalent dose at T1 or T2. There was no significant change between the T1 and T2 BZD doses within the groups. The diazepam equivalent dose at T1 was on average 26.2 mg (*SD *= 18.5) in the methadone group and at T2 26.5 (*SD *= 10.0); in the buprenorphine group, the respective values were 27.7 (*SD *= 24.1) and 21.0 (*SD *= 11.1). In other types of psychoactive drugs there were no statistically significant changes between the test points.

**Table 1:** Co-medications among patients used within the last 24 h before testing at T1 and T2

	**Methadone (*n *= 13)**		**Buprenorphine or Buprenorphine/Naloxone (*n *= 15)**	
	**Proportion of patients**	**Dose, range**	**Proportion of patients**	**Dose, range**

**Antidepressants (any), T1/T2**	**54%/46%**		**40%/53%**	
				
Citalopram T1	8%	40 mg	-	-
Citalopram T2	-	-	13%	10 mg
				
Escitalopram T1	8%	5 mg	-	-
Escitalopram T2	8%	10 mg	-	-
				
Doxepine T1	-	-	13%	75 – 100 mg
Doxepine T2	8%	50 mg	20%	25 – 100 mg
				
Fluoxetine T1	15%	20 – 30 mg	-	-
Fluoxetine T2	8%	40 mg	-	-
				
Milnacipran T1	-	-	-	-
Milnacipran T2	-	-	7%	50 mg
				
Mirtazapine T1	25%	15 – 30 mg	-	-
Mirtazapine T2	25%	30 mg	7%	30 mg
				
Paroxetine T1	8%	50 mg	7%	50 mg
Paroxetine T2	-	-	13%	40 mg
				
Sertraline T1	-	-	7%	50 mg
Sertraline T2	-	-	7%	50 mg
				
Trimipramine T1	-	-	-	-
Trimipramine T2	8%	150 mg	-	-
				
Venlafaxine T1	-	-	13%	75 mg
Venlafaxine T2	-	-	7%	75 mg

**Anxiolytics, sedatives and hypnotics: Benzodiazepines (any), T1/T2**	**87%/100%**		**93%/100%**	
				
Alpratzolam T1	-	-	13%	1 – 2 mg
Alpratzolam T2	-	-	13%	1 – 2 mg
				
Clonazepam T1	-	-	13%	2 – 5 mg
Clonazepam T2	-	-	-	-
				
Diazepam T1	46%	10 – 55 mg	47%	10 – 40 mg
Diazepam T2	38%	15 – 30 mg	67%	5 – 30 mg
				
Oxazepam T1	31%	60 – 120 mg	33%	30 – 90 mg
Oxazepam T2	46%	15 – 60 mg	20%	55 – 60 mg
				
MidazolamT1^a^	-	-	-	-
MidazolamT2^a^	-	-	7%	30 mg
				
Temazepam T1^a^	31%	20 mg	13%	20 mg
Temazepam T2^a^	15%	20 – 40 mg	13%	20 – 40 mg

**Non-benzodiazepine hypnotics (any), T1/T2**	**15%/8%**		**20%/7%**	
				
Zolpidem T1^a^	-	-	-	-
Zolpidem T2^a^	8%	10 mg	7%	10 mg
				
Zopiclone T1^a^	15%	7.5 mg	20%	7.5 – 15 mg
Zopiclone T2^a^	8%	7.5 mg	7%	7.5 mg

**Neuroleptics (any), T1/T2^b^**	**20%/8%**		**7%/7%**	
				
Levomepromazine T1	-	-	-	-
Levomepromazine T2	8%	50 mg	-	-
				
Quetiapine T1	20%	50 – 300 mg	7%	300 mg
Quetiapine T2	-	-	7%	150 mg
				
Rispiderone T1	-	-	-	-
Rispiderone T2	8%	-	-	-

**Substance abuse withdrawal symptom or (non-opioid) pain relievers (any), T1/T2**	**42%/25%**		**40%/13%**	
				
Disulfiram T2	-	-	-	-
Disulfiram T2	8%	600 mg	-	-
				
Hydroxyzine T1	25%	25 – 200 mg	27%	75 – 200 mg
Hydroxyzine T2	8%	50 mg	7%	100 mg
				
Ibuprofen T1	8%	600 mg	7%	400 mg
Ibuprofen T2	-	-	7%	600 mg
				
Lofexidine T1	8%	0,2 mg	20%	0.2 – 0.6 mg
Lofexidine T2	-	-	-	-
				
Metoclopramide T1	8%	10 mg	-	-
Metoclopramide T2	-	-	-	-
				
Naproxen T1	8%	500 mg	-	-
Naproxen T2	-	-	-	-
				
Propranol T1	8%	20 mg	-	-
Propranol T2	-	-	-	-
				
Valproate T1	8%	1000 mg	20%	500 – 1000 mg
Valproate T2	-	-	7%	100 mg

^a ^Used as a hypnotic on the night before testing.

^b ^Used for anxiolysis.

### Memory tests

**Working memory **refers to the limited capacity short-term store that temporarily maintains information, which is lost without rehearsal [28]. It was assessed by the Letter-Number-Sequencing task from the Wechsler Memory Scale-third version (WMS-III) and by the computerized version of the Paced Auditory Serial Addition Task (PASAT) from the FORAMENRehab software package [29-31].

**Immediate verbal memory **refers to immediate storage of verbally presented items in those situations that exceed the capacity of sensory-specific working memory stores. Typical examples of immediate verbal memory measures include recall of a list or story. Immediate verbal memory is thought to rely on both working and long-term memory stores. This concurrent activation of two memory stores has recently been experimentally confirmed [32]. Immediate verbal memory was assessed by two verbal memory tasks, a list learning and a story recall task: the Memory for Persons Data and The Logical Memory [29,33]. Both tasks were presented in modified versions. The details of these tasks are presented in our previous study [15].

**Memory consolidation **refers to the storage and consolidation of memory traces. Early memory consolidation lasts from minutes to hours and late memory consolidation from weeks to years; these rely partly on separate neural processes [34,35]. Early memory consolidation was assessed by the percentage of the Logical Memory and Memory for Persons Data items successfully recalled by free recall after a short delay (30 min). Late memory consolidation was assessed by free recall of the Memory for Persons Data items at T2, which occurred after at least four and on average six, months after initial learning. Participants were further asked to rate the certainty of their answers after the long delay. This may give additional information about the memory processes the participants are employing [36]. If the participant gave the right answer, it was asked if he/she was certain that he/she actually remembered the answer or if he/she only felt he/she knew the answer but was not certain about it. In the case of "felt" or no answer, three nearly identical alternatives were given, one of them being correct. After the participant gave his/her choice, he/she was asked if he/she remembered, felt, or just guessed the answer.

**Subjective memory **functioning was assessed by the Finnish version of the Memory Complaint Questionnaire, the MCQ [20]. In the MCQ, the participant is asked how his/her memory now functions compared to when he/she was younger. Several answers are given, using a Likert-type scale, describing how well memory functions in everyday tasks (remembering persons, things, news, shopping list items, etc.). A high score indicates subjective memory impairment.

### Statistical Analysis

Overall group differences in memory performance at T1 and T2 were tested for statistical significance using multiple planned analysis of covariance (ANCOVA) with years of education and verbal IQ estimate as covariates. Although there were no statistically significant differences between the groups in the verbal IQ, it was used as a covariate because it is known to affect memory performance in tasks with verbal content [37]. ANCOVA was followed, when appropriate, by pairwise group comparisons using normal comparison group as a reference group. The Holm's sequential Bonferroni procedure was used to control for Type I error across the pairwise comparisons [38]. In all analyses, statistical significance was set at 0.05 (two-tailed). In the Memory for Persons Data, the data were highly skewed due to a ceiling effect in the initial learning and recall at T1. At T2, the Memory for Persons Data for delayed recall was skewed because of small variance. Because of these violations of the assumptions of parametric testing, we analyzed these conditions by Kruskal-Wallis ANOVAs, which were followed, when appropriate, by pairwise Mann-Whitney *U *tests. In order to confirm the validity of combining buprenorphine/naloxone and buprenorphine-only patients, the ANCOVAs and ANOVAs were also performed with buprenorphine/naloxone patients (n = 12). The cross-sectional MCQ scores and the MCQ T2 differences between high vs. low score groups were analyzed by *t*-tests or Mann-Whitney *U *tests. Correlations between the MCQ values and cognitive variables were analyzed by Pearson's product moment correlation or Spearman's rho correlations depending on the normality of the variables. Correlations of at least .35 will be reported. The statistical significance of correlations was determined by using the Holm-Bonferroni procedure.

Longitudinal changes were analyzed by repeated measures ANCOVA using education and VIQ as a covariates and the comparison group as a reference group. All statistical analyses were performed using SPSS statistical software, version 15.0, with the exception of the effect size calculations. For this purpose, an effect size calculator provided by Durham University, UK was employed [39,40]; and the Cohen's d values were corrected by Hedge's correction for small sample bias.

## Results

### Study demographics

Table 2 shows the comparisons of demographic variables of each group. The group difference in verbal intelligence (Verbal IQ) was statistically non-significant even though the comparison group had more education than the patient groups. There were no significant differences between the OST groups in history of substance abuse, duration of OST, or the prevalence of psychiatric co-morbidity. Personality disorder diagnoses were common in both patient groups. Buprenorphine was the main opioid of abuse before the OST admission in both groups. Among patients, no major change in the number of non-opioid substances abused during the recent month before the T1 or T2 testing was seen during the study period.

**Table 2:** Group demographics

	**Methadone** **(*n *= 13)**	**Buprenorphine or Buprenorphine/Naloxone** **(*n *= 15)**	**Normal Comparison** **(*n *= 15)**	**Group comparison** ***p*-values^a^**
Age, mean of years at T1 (*SD*)	29.2 (6.8)	27.7 (6.8)	28.7 (9.6)	M vs. BN, *p *= 0.99
				M vs. NC, *p *= 0.99
				BN vs. NC, *p *= 0.99

Sex: females/males	7/6	4/11	8/7	M vs. BN, *p *= 0.14
				M vs. NC, *p *= 0.98
				BN vs. NC, *p *= 0.14

Verbal intelligence, Mean^b ^(*SD*)	100.6 (11.4)	99.4 (9.3)	104.1 (9.6)	M vs. BN, *p *= 0.99
				M vs. NC, *p *= 0.74
				BN vs. NC, *p *= 0.63

Education, mean of years (*SD*)	10.1 (1.2)	10.5 (2.0)	12.6 (1.3)	M vs. BN, *p *= 0.54
				M < NC***, *p *< 0.001
				BN < NC**, *p *= 0.006

Opioid of abuse used within last month at T1				
Buprenorphine	85%	100%	-	M vs. BN, *p *= 0.48^c^
Heroin	15%	0%		

Other substances of abuse used within last month at T1 and T2				
Alcohol (heavy use)^d^	15%/15%	13%/7%	7%/7%	M vs. BN vs. NC (T1/T2), *p *= 0.99^c^/0.99^c^
Amphetamine	8%/8%	13%/7%	-	M vs. BN vs. NC (T1/T2) *p *= 0.99^c^/0.99^c^
Benzodiazepine, any Use	100%/100%	100%/100%	0%/0%	M & BN > NC*** (T1/T2), *p *< 0.001^c^/*p *< 0.001^c^
Benzodiazepine, extra doses	38%/38%	42%/33%	-	M vs. BN (T1/T2), *p *= 0.62/*p *= 0.78^c^
Cannabis	31%/31%	40%/27%	-	M vs. BN (T1/T2), *p *= 0.83^c^/0.84^c^
Nicotine (daily use)	100%/100%	100%/100%	33%/33%	M & BN > NC*** (T1/T2), *p *< 0.001^c^/*p *< 0.001^c^

Duration of OST in the day of testing at T1, Mean of days (*SD*)	21 (14)	19 (12)	-	M vs. BN, *p *= 0.69

Duration of OST on the day of testing at T2, Mean of days (*SD*)	213 (25)	224 (17)	-	M vs. BN, *p *= 0.15

Participants with other dependence or abuse diagnosis at T1				
Alcohol	0%	0%	0%	M vs. BN vs. NC, *p *= 0.99^c^
Amphetamine	0%	0%	-	M vs. BN, *p *= 0.99^c^
Benzodiazepine	100%	100%	-	M vs. BN, *p *= 0.99^c^
Cannabis	15%	20%	-	M vs. BN, *p *= 0.99
Nicotine	100%	100%	33%	M vs. BN, *p *= 0.99^c^
				M vs. NC, *p *= 0.13^c^
				BN vs. NC, *p *= 0.12^c^

Participants with any DSM-IV axis I diagnosis at T1	15%	20%	0%	M vs. BN, *p *= 0.99^c^
				M vs. NC, *p *= 0.21^c^
				BN vs. NC, *p *= 0.22^c^

Participants with any personality disorder diagnosis (DSM-IV axis II) at T1	54%	59%	0%	M vs. BN, *p *= 0.99^c^
				M > NC**, *p *= 0.003^c^
				BN > NC**, *p *= 0.002^c^

Duration of opioid abuse at T1, Mean of years (*SD*)	11.4 (5.5)	9.0 (2.9)	-	M vs. BN, *p *= 0.26

Duration of any substance abuse at T1, Mean of years (*SD*)	15.0 (5.1)	13.4 (5.2)	-	M vs. BN, *p *= 0.37

^a ^Based on pairwise group comparisons with analysis of variance (ANOVA) or chi-squared test.

^b ^Estimation based on the WAIS-R Vocabulary score.

^c ^Fisher's Exact Test (2-tailed).

^d ^Alcohol use was considered heavy if it was at least a mean of 16 portions weekly for females and 24 portions weekly for males. One portion was defined as 12 g of alcohol.

BN = buprenorphine or buprenorphine/naloxone, M = methadone, NC = Normal comparison.

*** = statistically significant at level *p *< 0.001. ** = statistically significant at level *p *< 0.01. * = statistically significant at level *p *< 0.05.

### Group comparisons at T1

In Table 3, an overview of unadjusted memory test results at both test points is presented together with statistical comparisons for years of education and verbal IQ adjusted values, whenever adjusting was possible. In working memory tests, the methadone patients were inferior to controls in the PASAT, but in the Letter-Number Sequencing the group difference remained non-significant. The buprenorphine patients were inferior to normal comparison participants on the both of the working memory tests. In immediate verbal memory as measured by the first learning trial of the Memory for Persons Data, both patient groups performed significantly worse than normal comparison group. In early memory consolidation as measured by short-term retention of percentages of the Logical Memory and the Memory for Persons Data items, no significant group differences emerged. The statistical significance of the analyses remained the same when the analyses outlined in the Table 3 were done with buprenorphine/naloxone patients (n = 12) instead of combining the buprenorphine-only and buprenorphine/naloxone patients. Statistically significant values of overall group effects were, in order, The Letter-Number Sequencing, The PASAT, the first trial of the Memory for Persons Data, and the MCQ (*F *(2, 35) = 3.63, *p *= 0.009; *F *(2, 35) = 9.57, *p *< 0.001; *Χ*^2 ^(2, *N *= 40) = 7.99, exact *p *= 0.018; *Χ*^2^, (2, *N *= 40) = 11.83, exact *p *= 0.004). After this, pairwise analyses between the buprenorphine/naloxone and normal comparison groups were performed. In the Letter-Number Sequencing the pairwise group comparison was statistically non-significant *(t *(26) *= *2.67, *p *= 0.065). In the PASAT and in the first trial of the Memory Persons Data, the buprenorphine/naloxone group showed worse performance than the normal comparison group (*t *(26) = 4.71, *p *= 0.00; Mann-Whitney *U *= 45.50, exact *p *= 0.028, respectively). In the MCQ, the buprenorphine/naloxone patients reported more memory complaints than the comparison participants (Mann-Whitney *U *= 26.00, exact *p *= 0.004).

**Table 3:** Group comparisons of memory functions at T1 and T2

**Domain or Test**	**Methadone** **(*n *= 13)**	**Buprenorphine or Buprenorphine/Naloxone** **(*n *= 15)**	**Normal Comparison** **(*n *= 15)**	**Group effect**	**Statistical comparisons between normal comparison and patient groups using years of education and VIQ adjusted scores, whenever possible^b^**	**Effect sizes, whenever possible**
	*M (SD)*	*M (SD)*	*M (SD)*			

**Working memory, raw scores**						

WMS-III Letter-Number Sequencing at T1	9.6 (2.3)	8.4 (2.3)	11.7 (3.2)	*F *(2, 38) = 4.57 *p *= 0.017*	*t*(27) = 1..84, *p *= 0.074, M vs. NC = *ns*	d = 0.68
					*t(29) = *3.02, *p *= 0.008, NC > BN **	d = 1.01
WMS-III Letter-Number Sequencing at T2	8.6 (2.1)	9.2 (2.3)	11.6 (2.9)	*F (2, 38) *= 4.19, *p *= 0.023*	*t*(27) = 2.76, *p *= 0.018, NC > M*	d = 1.05
					*t *(29) = 2.39, *p *= 0.022, NC > BN *	d = 0.83

PASAT at T1	31.4 (9.2)	31.8 (10.7)^a^	46.7 (9.4)	*F (2, 38) *= 9.84 *p *= 0.001***	*t*(27) = 4.19, *p *< 0.001, NC > M ***	d = 1.43
PASAT at T2	31.6 (8.6)	34.1 (8.4)	46.0 (8.7)^a^	*F (2, 38) *= 7.15 *p *= 0.002**	*t*(29) = 3.70, *p *< 0.001, NC > BN***	d = 1.54
					*t*(27) = 3.47, *p *= 0.002, NC > M**	d = 1.42
					*t*(29) = 3.32, *p *= 0.002, NC > BN**	d = 1.24

**Immediate verbal memory, raw scores**						

Memory for Persons Data, first trial at T1	10.7 (2.6)	10.8 (2.5)	13.0 (1.5)	χ^2^(2, *N *= 43) = 8.91 *p *= 0.012*	*U *= 43.0, *p *= 0.011, NC > M*	-
					*U *= 51.5, *p *= 0.020, NC > BN*	

Memory for Persons Data, sum of last two trials (T1)	14.9 (0.2)	14.6 (0.7)	14.9 (0.2)	χ^2 ^(2, *N *= 43) = 2.75 *p *= 0.25	*-*	-

WMS-III Logical Memory, immediate free recall (T1)	12.9 (2.4)	15.1 (4.3)	16.3 (3.6)	*F (2, 38) *= 1.90, *p = *0.16	-	-
WMS-III Logical Memory, immediate free recall (T2)	14.2 (3.1)	14.1 (3.3)	16.3 (3.1)	*F (2, 38) *= 1.25, *p *= 0.30	-	-

**Memory consolidation, percentages**						

WMS-III Logical Memory, free recall after short-delay (30 min) (T1)	91.4 (15.3)	91.7 (14.0)	87.5 (13.1)	*F (2, 38) *= 0.64, *p *= 0.94	-	-
WMS-III Logical Memory, free recall after short (30 min) delay (T2)	87.1 (14.4)	93 .8 (17.1)	98.3 (14.1)	*F (2, 38) *= 1.28, *p *= 0.29	-	-

Memory for Persons Data, free recall after short delay (30 min) (T1)	92.8 (7.9)	98.2 (6.1)	98.7 (3.1)	χ^2^(2, *N *= 43) = 4.48 *p *= 0.11	-	-

Memory for Persons Data, free recall after long delay (4 – 8 mo) (T2)	22.1 (18.1)	29.8 (23.2)	32.4 (22.1)	χ^2^(2, *N *= 43) = 1.54 *p *= 0.46	-	-

Memory for Persons Data, recognition after long delay (4 – 8 mo) (T2)	79.6 (10.6)	82.1 (12.9)	81.3 (10.7)	*F (2, 38) *= 0.60 *p *= 0.55	-	-

**Memory complaints, raw score**						

The Memory Complaint Questionnaire (T1)	26.6 (5.7)	26.0 (5.4)	20.4 (2.5)	χ^2^(2, *N *= 43) = 11.25	*U *= 39.0, *p *= 0.012, NC < M*	-
				*p *= 0.004**	*U *= 40.0, *p *= 0.008, NC < BN **	-
The Memory Complaint Questionnaire (T2)	25.6 (3.2)	24.5 (6.7)	20.4 (1.5)	χ^2 ^(2, *N *= 43) = 14.04	*U *= 16.5, *p *< 0.001, NC < M***	
				*p *= 0.001***	*U *= 49.0, *p *= 0.015, NC < BN *	

Note: PASAT = Paced Auditory Serial Addition Task;

WMS-III = Wechsler Memory Scale-third version.

BN = buprenorphine or buprenorphine/naloxone, M = methadone, NC = Normal comparison.

^a ^= Missing value of one participant was substituted by carry-over value from the first test.

*** = statistically significant at level *p *< 0.001, ** = statistically significant at level *p *< 0.01, * = statistically significant at level p < 0.05, *ns *= non-significant.

### Group comparisons at T2

At T2, both patient groups were inferior to normal comparison group in working memory tests. In immediate verbal memory assessed by the immediate recall of the Logical Memory items, no significant group differences were seen. In early memory consolidation assessed by the Logical Memory short-term retention percentage, group differences were not significant. In late memory consolidation assessed by the Memory for Persons Data free recall or recognition retention percentages after at least four months' delay, there were no group differences. Again, dropping buprenorphine-only patients from the buprenorphine group did not change the statistical significance of the overall ANOVAs or ANCOVAs. Significance values of overall group effects were, in order, the Letter-Number Sequencing, The PASAT, and the MCQ (*F*(2, 35) = 3.82, *p *= 0.032, (*F*(2, 35) = 7.52, *p *= 0.02, (*Χ*^2^, (2, *N *= 40) = 15.91, exact *p *< 0.001). Pairwise comparisons between the buprenorphine/naloxone and normal comparison groups favored the comparison group in both working memory tasks: the Letter-Number Sequencing and The PASAT, respectively (*t *(26) = 2.21, *p *= 0.03; *t *(26) = 3.33, *p *= 0.002). In the MCQ, the buprenorphine/naloxone patients reported more memory complaints the normal comparison participants (Mann-Whitney *U *= 29.50, exact *p *= 0.004).

**Figure 1:**
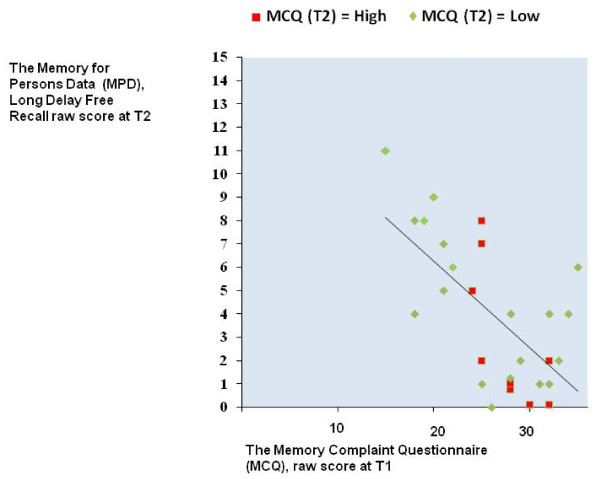
**Correlation percentage of the Memory for Persons Data, delayed recall (T2) scores by group**.

Interestingly, total "black-outs" in long delay free recall were rare. Only one methadone patient, two buprenorphine patients, and one comparison participant could not recall any items from the Memory for Persons Data in this condition. From Figure 1, which shows lines for cumulative percentages, it can be seen that about 50% of the normal comparison participants and buprenorphine patients could recall at least 4 items out of 15 correctly, while the corresponding score was 2 items among the methadone patients. When asked about the certainty of their answers, the patients were non-significantly more certain than the normal comparison participants that they actually remembered, not just felt, the correct answers they gave. On average, methadone-treated patients said that they surely remembered a mean of 64.1% of their correct free recall answers (*SD *= 38.8). In the buprenorphine group the corresponding figure was 67.2% (*SD *= 24.1) and in the normal comparison group 45.5% (*SD *= 31.3). In the same vein, there were no significant group differences in certainty of recognized correct answers (data not shown). Both patient groups again reported significantly more memory complaints in the MCQ.

### Correlations between subjective and objective memory functions among the patients

**Figure 2:**
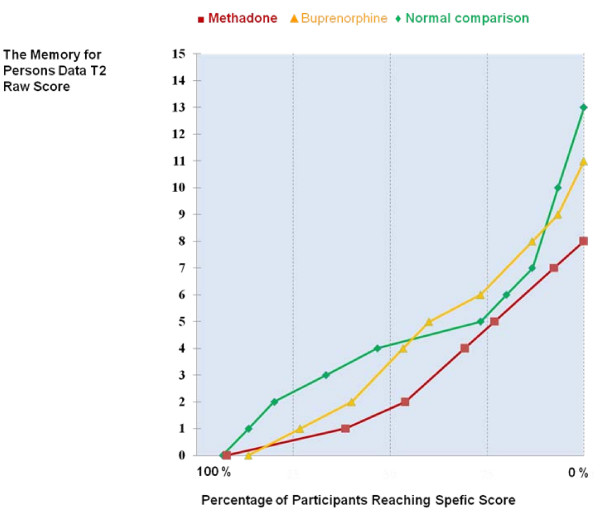
Cumulative percentage of the Memory for Persons Data, delayed recall (T2) scores by group.

The highest correlation between subjective MCQ score and the objective memory tests completed by the OST patients at T1 was – .38 for the Logical Memory retention percent after short delay (30 min). However, after correction for multiple comparisons, this moderate correlation was statistically non-significant. The correlation between the MCQ score at T1 and the long delay free recall of the Memory for Persons Data items at T2, that is, at least four months after initial learning, was – .58 and statistically significant, *p *= 0.028. This relationship is depicted in Figure 1. At T2, two moderate correlations between subjective MCQ score at T2 and objective memory performance of the patients were seen: – .40 for long delay free recall of the Memory for Persons Data items and – .39 for the PASAT. However, after correction for multiple comparisons these were no longer statistically significant. In order to explore how the OST patients with high MCQ scores at the stabilized phase (T2) are different from those with low MCQ scores, the patient group was divided into high vs. low memory complaints groups using the T2 MCQ median score as the cut-off. Patients with scores of 26 or more at T2 made up the high memory complaints group (*n *= 14) and those with scores up to 25 the low memory complaints group (*n *= 14). There were no statistically significant differences between the high and low memory complaint groups in demographics, substance abuse history, or treatment or medication variables. For cognitive variables, there were no significant differences between the groups except on the measure of Memory for Persons Data free recall, on which the respective means for the high and low groups were 16.7% (*SD *= 16.7) and 35.7% (*SD *= 21.0); (*t *(27) = 2.66, *p *= 0.013). As can be seen from Figure 2, most of the patients classified as high memory complainers at T2 already had high MCQ scores at T1. Seventy-one percent of the high memory complainers at T2 complained of memory problems at T1 matching or exceeding the MCQ high memory complaints cut-off score of 26.

### Longitudinal changes

In the first working memory task, the Letter-Number Sequencing, methadone-treated patients' performance seemed to deteriorate from T1 to T2 as shown by a decrease in their raw scores. On the other hand, they seemed to improve in immediate verbal memory performance. Opposite trends were seen in the buprenorphine group. However, no significant group by time interactions emerged.

## Discussion

The main finding of this longitudinal study is the persistence of the working memory deficit in OD patients treated with methadone or buprenorphine along with BZDs. At T1, the buprenorphine patients were inferior to normal comparison participants in both working memory tests; and the methadone patients performed worse than normal comparison participants at the second working memory task, the PASAT. At T2, both patient groups were impaired relative to a normal comparison group on both working memory tests. The working memory tests used in this study have both been used also earlier in opioid-related neuropsychological studies. In an earlier study by Verdejo-Garcia et al., minimum 15 days abstinent heroin abusers outperformed methadone-treated OST patients on our first working memory measure, the Letter-Number Sequencing [41]. In a study by Mintzer and Stitzer, methadone-treated OST patients performed worse than a well-matched normal comparison group on a two-back working memory task closely resembling the PASAT [42]. The evidence for opioid agonist effects is not, however, unambiguous because in both of these studies, the OST patients had a previous history of using other substances of abuse, including BZDs. On the other hand, in a study by Sjögren et al., pain patients treated with pain drugs other than opioids outperformed non-addicted opioid-treated pain patients on the PASAT [17]. In the same vein, a recent study showed that the opioid agonist morphine negatively affects working memory performance in healthy volunteers [16]. Although pure OST drug effects on working memory seem possible, the effects of OST drugs, alone or in combination with BZDs, on working memory can only be reliably examined if OST patients with and without a history of BZD use can be compared.

Our hypothesis of impaired performance in immediate verbal memory was partially confirmed as both patient groups were impaired at T1 in the first trial of a list learning task, the Memory for Persons Data. This finding is in line with earlier studies showing similar deficits among methadone patients [9,43]. However, the stability of this deficit in verbal list learning remains to be studied because the Memory for Persons Data learning task was not repeated at T2. Of note here is the study of Gruber et al. concerning an earlier treatment phase than was investigated in our study [14]. In their study methadone-treated patients' verbal list learning performance, improved between the first testing performed after a mean of two weeks of OST and the second after two months of treatment. However, a control group was lacking in their study. Although alternate test forms were used, practice effect in repeated verbal memory testing cannot be ruled out [44]. Thus, the evidence for early improvement of memory function is not strong.

Buprenorphine patients with concurrent BZD medication showed inferior list learning during early OST (T1). This finding is in line with a recent study by Soyka et al. in which buprenorphine patients without other dependencies were also inferior to normal comparison participants in verbal learning [45]. In a recent study by Loeber et al. no significant correlation was found between buprenorphine dose and verbal list learning performance [46]. On the other hand, Lintzeris et al. have reported that buprenorphine in combination with the BZD diazepam impairs delayed verbal memory more than buprenorphine given alone [19]. In sum, further studies of the possible "pure" buprenorphine effects or the additive negative effects of buprenorphine and BZDs on immediate verbal memory are needed.

Memory consolidation was examined by short- and long-term retention percentages. No significant group differences between patient groups and normal comparison group were observed in any condition. This is surprising because mu opioid receptor agonists and BZDs are known to negatively affect memory consolidation [47-50], However, our study is the first to study memory consolidation up to late memory consolidation that starts few hours after event occurrence [34,35]. Further studies are needed to examine if the observation of no memory consolidation impairment among OD patients is due to development of tolerance to negative effects of these drugs. There is some evidence for tolerance to methadone's long-term effects on episodic memory [51]. Tolerance for episodic memory impairing effects of BZDs, in general, are small [52], but among young individuals development of tolerance has been reported [53]. The second possible explanation for no memory consolidation impairment is that negative effects of opioids given along with BZDs may be hard to detect without a change in drug status. This means a change from a relatively highly drugged state to a low or non-drugged state or reverse. It has been reported that state change from BZD drug to placebo condition may negatively affect on memory retrieval in comparison to continuous BZD condition [54].

Analyses of long-term memory consolidation showed that among OST patients those with high memory complaints at T2 performed worse than those with low memory complaints in late memory consolidation assessed by free recall of the Memory for Persons Data items after a mean delay of six months. Of note here is the observation that there were no significant differences between high and low memory complainers on any background or other cognitive variables.

Self-rated memory problems were elevated among OD patients treated along with BZDs at both test points. Thus the patients feel that in regards to memory function their quality of life does not improve during the OST. Although OD patients often have both subjective and objective memory problems, few studies have addressed the relationship between subjective and objective memory function among patients with substance abuse problems [13,55,56]. In these studies patients' memory complaints have had small, if any, associations with their objective memory performance. In our study, though, moderate relationships were seen between subjective memory complaints and objective memory test performance, especially in late memory consolidation. Unfortunately, late memory consolidation deficit is not easily captured by standard neuropsychological assessment.

Methodological innovations to assess long-term memory consolidation in clinical settings are needed.

### Treatment and policy implications

Working memory function is considered a gateway for problem solving in new situations, which requires fluid intelligence and executive function. Thus, when working memory capacity is low, practical reasoning tends to result in instant firm decisions that are based on readily available salient observations [57,58]. Among OST patients this may mean that individuals with low working memory capacity readily associate their negative sentiments with the common belief that their OST medication is "insufficient". They may feel overwhelmed if asked to consider the counterexamples that co-occurrence of medication and negative sentiments may be coincidental or that negative drug effects may be short-lived in comparison to the positive effects that will show up later.

An OST patient who is using BZD medication and who has working memory impairment may show excellent memory in one instance and very poor memory in another. The variability of a patient's performance level in rehabilitation settings or at work or school may cause confusion in the clinic and the community. To minimize this, adequate examinations should be performed, and information should be provided to the patient and his/her treatment team more frequently than is currently the case.

The results indicate that memory deficits in OST patients with current or recent BZD use are rather stable at least during the first six months of their treatment. It is possible that this is associated with OST drugs and BZDs given legally to the OD patients. However, this does not mean that OST would be harmful for the recovery of OD patients. OD patients entering OST are, in general, so stuck in the addiction, that a abstinence oriented treatment program with no opioid or BZD agonists is a realistic alternative only in rare cases [1,4]. Both treatment alternatives are needed, but OST should be seen as the mainstream option.

### Limitations

Comparing a clinical sample of OST patients who use BZDs and other psychoactive medications against normal comparison participants imposes several limitations. Some of the patients (see Table 2), but none of the comparison participants were abusing illicit drugs. This is clear confounding factor that is difficult to eliminate when evaluating performances in memory tests. The same applies to other psychoactive medications that were legally given to some of the patients but none of the comparison participants. Thus, our results cannot be generalized to OD patients without psychoactive medications who have achieved long-term abstinence from any illicit use of drugs. Psychiatric comorbidity that included Axis I and Axis II disorders was common among patients and absent among comparison participants. A recent study by Prosser et al [59] examined correlates of cognitive function in a relatively large sample of opioid dependent patients (n = 56). It was found that personality pathology accounted for a greater portion of the variance in cognitive performance than any of the variables of drug use history. However, the only memory variable included in their analyses was immediate visual memory.

The mean opioid agonist doses given to our patients changed between test points, while the mean BZD doses and illegal substance abuse remained rather stable. The methadone dose increased from a mean of 73 mg at T1 to 126 mg at T2. The buprenorphine dose increased from a mean of 17 mg at T1 to 23 mg at T2. Thus, dose change and time factors are both affecting the results, and with our study design, separating these effects is not possible. The buprenorphine group included both buprenorphine-only and buprenorphine/naloxone patients. This was partially a practical issue because the majority of buprenorphine patients in Finland have been transferred to buprenorphine-naloxone combination medication. There is no evidence that sublingual naloxone exhibits opioid antagonist activity or would interfere with the opioid agonist effects of buprenorphine [26,60]. However, because there are no studies directly comparing buprenorphine-only and buprenorphine/naloxone patients, combining these patients can be considered a limitation of our study. The list learning task (the Memory for Persons Data) was not repeated at T2, which poses a limitation for the analyses of immediate verbal memory. Psychoactive drugs, such as short-acting non-BZDs, neuroleptics, or opioid withdrawal relievers, were given to both patient groups in order to alleviate opioid withdrawal symptoms or to treat psychiatric comorbidity. The possible interactions of OST medications with these medications warrant further studies with larger sample sizes. Recent-month drug screens were considered important because it is known that long-term use of benzodiazepines or cannabis may have a negative impact on cognitive function even weeks after cessation of use [18,61]. However, our data cannot determine the precise doses used during the recent month, nor does the data cover full time span of the follow-up. Thus, the results do not reflect "pure" drug effects of OST drugs and BZDs. On the other hand, no major differences between the substance abuse profiles of methadone and buprenorphine patients were seen. OD patients may differ from the general population already in their premorbid cognitive functioning [62]. Screening for premorbid conduct or attention deficit disorder could possibly reveal interactions with current cognitive functions among OST patients [63,64]. However, retrospective assessment of these has low reliability in the absence of longitudinal records [65]; therefore, these assessments were not done in our study. Finally, our sample size was relatively small, and therefore type 2 errors cannot be excluded.

## Conclusion

OD patients treated with methadone or buprenorphine along with BZDs showed substantial deficits in working memory both during beginning of the treatment, and after six months of treatment. Given the previously stated limitations of this study, we conclude that OD patients taking opioid agonist drugs and BZDs score worse than normal comparison persons in tests of memory during first six months of their OST. Thus, it is possible that the working memory deficit observed among these patients might be relatively permanent. An immediate verbal memory deficit may also be seen among them. Surprisingly, there were no significant memory consolidation differences between the patient groups and normal comparison group. On the other hand, OST patients reported subjective memory problems that were associated with poor late memory consolidation. This has obvious functional relevance for the patients. Therefore, we propose that the relationship between subjective and objective memory function should be taken into account in longitudinal studies of substance abuse treatment and clinical practice.

## Competing interests

The authors declare that they have no competing interests.

## Authors' contributions

PR planned and performed cognitive testing and statistical analysis. He wrote the first version of the manuscript and prepared the final manuscript. HA conceived the idea of the study and advised in manuscript preparation. HK participated in the design of the study and in manuscript preparation. CF carried out psychiatric investigations. All authors prepared, read and accepted the final manuscript.
